# Neuromuscular Complications of Targeted Anticancer Agents: Can Tyrosine Kinase Inhibitors Induce Myasthenia Gravis? Getting Answers From a Case Report up to a Systematic Review

**DOI:** 10.3389/fonc.2021.727010

**Published:** 2021-10-15

**Authors:** Dimitrios C. Ziogas, Dimitrios Mandellos, Charalampos Theocharopoulos, Panagiotis-Petros Lialios, Spyros Bouros, Paolo A. Ascierto, Helen Gogas

**Affiliations:** ^1^ First Department of Medicine, National and Kapodistrian University of Athens, School of Medicine, Laiko General Hospital, Athens, Greece; ^2^ Department of Neurology, Hygeia Hospital, Athens, Greece; ^3^ Department of Melanoma, Cancer Immunotherapy and Development Therapeutics, Istituto Nazionale Tumori IRCCS Fondazione Pascale, Napoli, Italy

**Keywords:** TKIs (tyrosine kinase inhibitors), metastatic cancer, myasthenia (myasthenia gravis—MG), targeted therapies, case report, review—systematic

## Abstract

More than 40 tyrosine kinase inhibitors (TKIs) have received hematological or oncological indications over the past 20 years, following the approval of imatinib, and many others are currently being tested in clinical and preclinical level. Beyond their common toxicities, no certain agent from this large class of molecularly targeted therapies was strongly associated with “off-target” impairment of neuromuscular transmission, and although myasthenia gravis (MG) is a well-characterized autoimmune disorder, only few sporadic events proven by serologically detected causative autoantibodies and/or by positive electrophysiological tests are reported in the literature. Herein, we present the first case of anti-MUSK (+) MG in a woman with metastatic BRAF-mutant melanoma after long-term treatment with dabrafenib (BRAF inhibitor) and trametinib (MEK inhibitor). Triggered by this report, a systematic literature review was conducted, summarizing all other cancer cases that developed MG, after exposure to any type of targeted agent and regardless of the underlying malignancy. All available data on the clinical diagnosis, the potential of administered TKIs to induce a seropositive myasthenic syndrome, the immune and non-immune-mediated pathogenesis of postsynaptic damage, and the challenging management of this neuromuscular toxicity were collected and discussed. In the presented case, MG was confirmed by both autoantibodies and nerve-conduction tests, while its reactivation after TKIs rechallenge supports a more than coincidental association. The following review identified 12 cancer cases with TKI-related MG in six case reports and one case series. In most of them, the myasthenia diagnosis was challenging, since the clinical symptomatology of fatigable weakness was not corroborating with consistent laboratory and electrophysiological findings. In fact, anti-AchR titers were positive in five and anti-MuSK only in the abovementioned individual. The symptomatology corresponded to TKI discontinuation and standard treatment with pyridostigmine and prednisolone; intravenous immunoglobulin was added only in three, and two required mechanical ventilation. In an era where TKIs will be prescribed more frequently for various malignancies, even in combinations with immune-checkpoint inhibitors, this report synthesizes their risk for neuromuscular complications and increases the clinicians’ awareness in order to extend the on-treatment and overall survival of TKI-treated cancer patients.

## Introduction

Myasthenia gravis (MG) is induced by an autoantibody-mediated reaction against functional postsynaptic components of the neuromuscular junction [e.g., acetylcholine receptor (AchR), muscle-specific kinase (MuSK), and lipoprotein receptor-related protein 4 (LRP4)] (1). The resulting impairment of postsynaptic status leads to fluctuating degree of fatigable weakness: (i) only in the eyelids and extraocular muscles (ocular myasthenia) or (ii) more often including ocular, bulbar, limb, and respiratory muscles (generalized myasthenia). The clinical diagnosis of MG can be confirmed by the serological detection of these autoantibodies and by the electrophysiological testing of nerve-to-muscle conduction [e.g., repetitive nerve stimulation (RNS) and single-fiber electromyography (EMG)] (1). Few neoplasms are associated with MG with thymoma being the most common among them (10–15%) (2), while many drugs of everyday clinical practice have been accused to cause or unmask myasthenic manifestations, such as anesthetic agents, antibiotics or antivirals (e.g., aminoglycosides, fluoroquinolones, macrolides, or antiretrovirals) (3), anticonvulsants (e.g., gabapentin, phenobarbital, and phenytoin), statins (e.g., atorvastatin, pravastatin, rosuvastatin, and simvastatin) (3), psychiatric drugs (e.g., haloperidol, chlorpromazine, and prochlorperazine), and other drugs such as D-penicillamine (4), chemotherapies (e.g., cisplatin, fludarabine) even new immune checkpoint inhibitors such as nivolumab, pembrolizumab, and ipilimumab (5–7). However, all these abovementioned medications have been pinpointed in low evidence level studies and with no clear pathophysiological mechanisms.

Similarly, limited agents from the large class of molecularly targeted anticancer tyrosine kinase inhibitors (TKIs) have also been reported, up to now, to rise causative autoantibodies in the serum and to adversely affect neuromuscular transmission. TKIs bind reversibly (non-covalently) or irreversibly (covalently) to the active or inactive conformations of the corresponding kinases (e.g., the ATP-binding domain, the substrate binding site, or the allosteric sites outside the ATP pocket) and inhibit these kinases from phosphorylating tyrosine residues of their substrates and from activating subsequent downstream pathways (8–10). Despite their similar functional principle, there are benefits and drawbacks associated with each one of their mechanisms of action (11). These pharmaceutical compounds have diverse kinase affinity, specificity, and selectivity, and may block a broader than expected range of targets, offering “off-target” toxicity (12, 13). Following the approval of imatinib in 2001, more than 40 TKIs have received hematological or oncological indications over the past 20 years, and many others are currently being tested in clinical and preclinical level, contributing to precision cancer medicine according to individual genetic alterations (14). Beyond their common toxicities (e.g., diarrhea, rash, hypertension, elevated transaminases, and fever), targeted agents have also been associated with few rare, sporadically described in the literature, but potentially severe, “off-target” sequelae, including myasthenic syndrome (15–21). Herein, we present the first patient with metastatic melanoma who developed anti-MuSK(+) MG after long-term administration of BRAF/MEK TKIs. The drug-induced etiology of this complication was confirmed by the rapid relapse of myasthenic symptoms after the resumption of dabrafenib and trametinib. Beginning from this case, the following literature review collects all currently published cancer cases that developed serologically and/or neurophysiologically proven MG under any type of TKI treatment and regardless of the underlying malignancy; and thoroughly discusses the entire management to overcome neuromuscular symptomatology.

## Case Presentation

**Figure 1:**
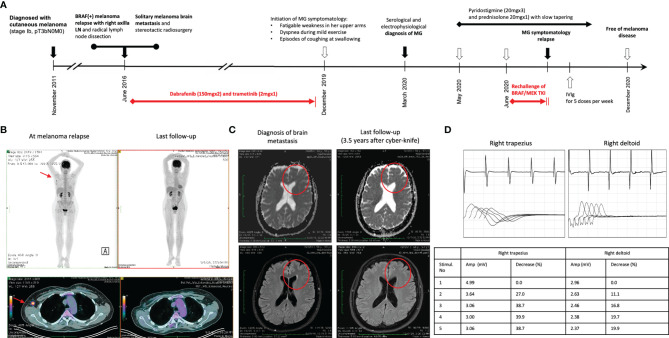
**(A)** Timeline of therapy and disease status for both melanoma and MG. **(B)** PET/CT findings at melanoma relapse and at the last follow-up. **(C)** MRI findings at melanoma brain metastasis diagnosis and at 3.5 years after stereotactic radiosurgery. **(D)** The results of RNS test in the right deltoid muscle and the right trapezoid muscle at the time of clinical presentation of myasthenia gravis.

A 57-year-old Caucasian woman, living in Athens with her husband, with no past medical history was diagnosed in November 2011 with a stage Ib cutaneous melanoma at her chest wall (pT3bN0, Breslow 3.65 mm, Clark IV, with presence of ulceration) and negative sentinel lymph node dissection (SLND) (0 of 5) in her right axilla (**Figure 1A**). Remaining at follow-up, 5 years later, imaging by whole-body computerized tomography (CT) revealed only a slightly enlarged lymph node in her right axilla, and she underwent to a positron emission tomography/CT scan (PET/CT) in June 2016 to confirm the activity and the extent of her disease. PET/CT scan showed abnormal hypermetabolic activity of this axillary lymph node lesion (maximum standardized uptake value, SUVmax = 9.4) with no evidence of active disease elsewhere (**Figure 1B**). Therefore, a radical lymph node dissection of her right axilla was directly performed. The histological examination detected only one lymph node infiltrated by melanoma cells, and molecular analysis showed that her melanoma harbored the *BRAF* mutation (c.1798_1799GT>AA) pV600K in exon 15. Soon after that, adjuvant therapy with high-dose interferon was administered, according to the established guidelines then. Due to the BRAF (+) nature of melanoma, a magnetic resonance of her brain was scheduled at the end of the same month, revealing a synchronous small solitary melanoma metastasis in her left lobe (**Figure 1C**). A stereotactic radiosurgery was performed to control brain involvement, and the patient immediately initiated targeted therapy with BRAF and MEK TKIs. She was already receiving dabrafenib (150 mg, every 12 h) and trametinib (2 mg, once daily) for approximately 3 years with good tolerance and no evidence of melanoma relapse, when she started to complain for fatigable weakness in her upper arms, gradually worsening in last 2 months with a parallel feeling of dyspnea during mild exercise and episodes of coughing at swallowing. A neurological consultancy was requested, and the physical examination identified serious bilateral proximal muscle weakness of the upper arms, no weakness of the lower limbs, and normal sensory findings. The repeated brain MRI recognized the almost resolved single lesion in her left lobe, and the MRI of the cervical spine showed an old intervertebral disk herniation. Chest CT had no evidence of thymoma, and her laboratory results revealed normal creatinine kinase (CK), thyroid-stimulating hormone (TSH), serum lactate, electrolytes, and erythrocyte sedimentation rate *(*ESR). In April 2020, the serological tests recognized no antibodies against AchR and voltage-gated calcium channel (VGCC) but high titers of anti-MuSK antibodies (21 nM; reference range, 0.015–0.030 nM). In order to confirm the diagnosis of seropositive MG, the patient underwent repetitive nerve stimulation (RNS). The RNS testing incorporates the surface stimulation and recording procedures used in routine motor nerve conduction studies and is abnormal in up to 70% of patients with generalized MG. During low-frequency (2–3 Hz) RNS, Ach becomes depleted at the examined neuromuscular junctions, and therefore, less is available for immediate release, resulting in smaller excitatory postsynaptic potentials (22). In patients without MG, all excitatory postsynaptic potentials exceed the threshold to generate an action potential, and no change in the summated compound muscle action potential is noted. In patients with MG, the number of AchRs is reduced, lowering the safety factor, and thus, during RNS, some excitatory postsynaptic potentials may not reach threshold, which means that no action potential is generated (22). This results in a decrement of the compound muscle action potential of 10% or more. In our case, the RNS test was totally consistent for MG, with decrease in motor voltage by 39% in the right deltoid muscle and with decrease in motor voltage by 22% in the right trapezoid muscle (**Figure 1D**). Although RNS is a sensitive test for MG, the patient underwent additionally an EMG, which was normal, to exclude a variety of other disorders that can also cause a decremental RNS response (e.g., myotonic disorders, severe myopathies, and Lambert–Eaton myasthenic syndrome) (22).

Treatment with pyridostigmine 20 mg three times per day and prednisolone 20 mg once daily was initiated in May 2020 with partial improvement after 4 weeks. Few days later, the BRAF/MEK inhibitors were resumed, but the myasthenic symptoms worsened again. Then, targeted therapy with dabrafenib/trametinib was permanently discontinued, and prednisolone was increased to 25 mg once daily. Due to persistence of symptomatology, our multidisciplinary team made the decision to concomitantly administer intravenous immunoglobulin (IVIg) for five consecutive doses per week, improving significantly her symptoms in the next 2 weeks. Keeping a slow tapering of prednisolone, the patient symptomatology was substantially resolved over the following months, and she remained under follow-up every 3 months with no further antimelanoma treatment. In December 2020, her anti-MuSK autoantibodies were still high but substantially decreased (15 nM; reference range, 0.015–0.030 nM). The new melanoma restaging is still pending, and the patient remains willing to receive again targeted therapy if it is needed in the future.

## Search Strategy—Data Extraction

**Figure 2:**
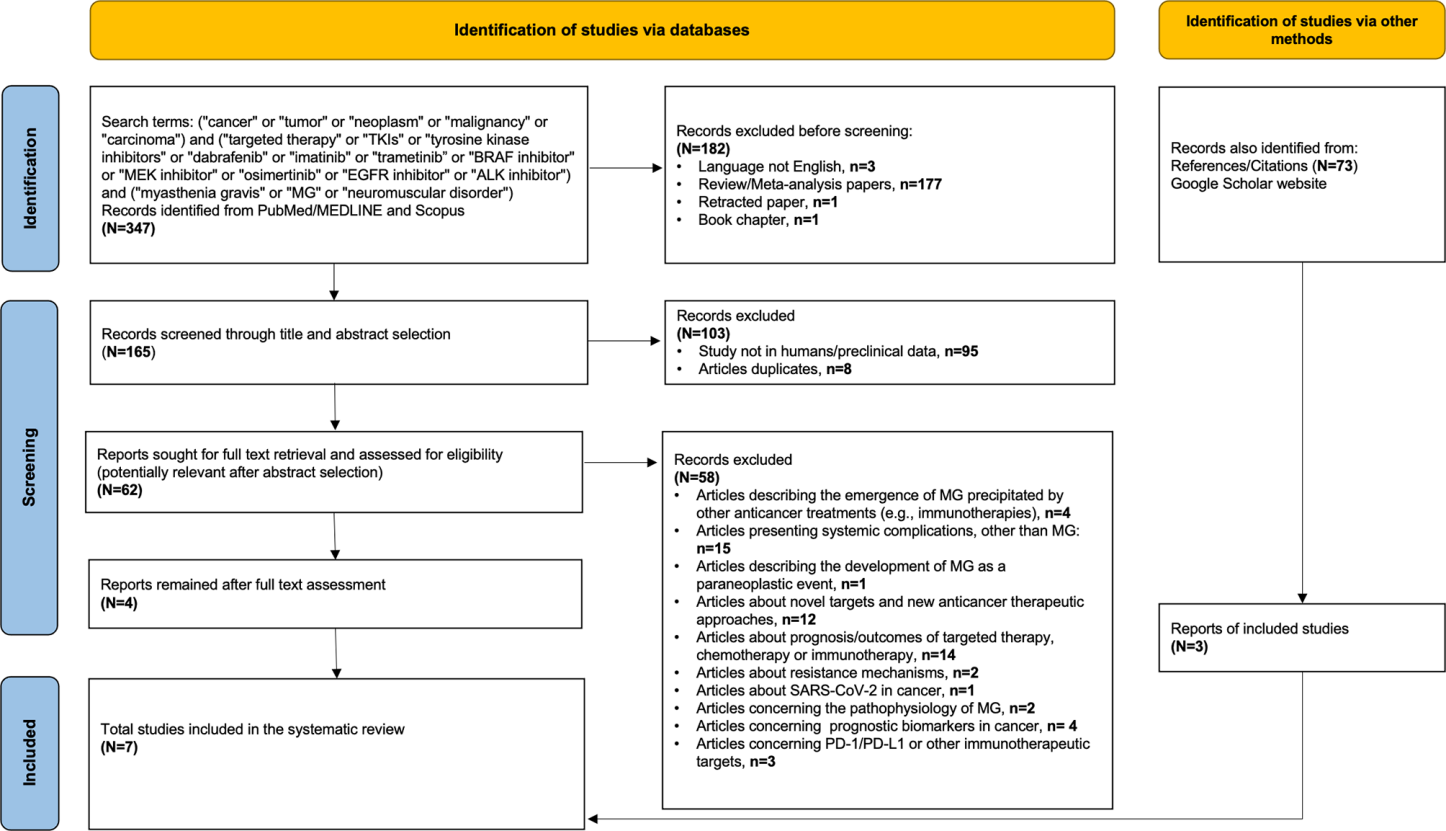
Flow diagram of literature search strategy, according to the Preferred Reporting Items for Systematic Reviews and Meta-analysis (PRISMA) 2020 guidelines.

In order to identify additional cancer cases with TKI-induced MG, we searched PubMed/MEDLINE and Scopus using the following terms: (i) terms suggestive of cancer (e.g., cancer, tumor, and malignancy), (ii) terms suggestive of targeted therapy [e.g., TKIs, tyrosine kinase inhibitors, BRAF/MEK inhibitors, epidermal growth factor receptor (EGFR) inhibitor, and anaplastic lymphoma kinase (ALK) inhibitor] including the most commonly used TKIs in each class (for example, dabrafenib, trametinib, imatinib, osimertinib, and alectinib), and (iii) terms suggestive of myasthenia gravis (e.g., MG, myasthenia gravis, neuromuscular disorder). The exact keywords used in both PubMed/MEDLINE and Scopus were the following: (“cancer” or “tumor” or “neoplasm” or “malignancy” or “carcinoma”) and (“targeted therapy” or “TKIs” or “tyrosine kinase inhibitors” or “dabrafenib” or “imatinib” or “trametinib” or “BRAF inhibitor” or “MEK inhibitor” or “osimertinib” or “EGFR inhibitor” or “ALK inhibitor”) and (“myasthenia gravis” or “MG” or “neuromuscular disorder”). Our search was up to September 15, 2021, with no specific start point and was limited only in papers written in English language. All studies (e.g., case reports, case series, and cohort studies) that described the presentation of MG after exposure to any targeted anticancer TKI agent in adult patients (e.g., age >18 years old) with any type of underlying solid or hematological malignancy were considered eligible. Reports describing the emergence of MG on other anticancer treatments (e.g., immunotherapies), studies presenting neurological or neuromuscular complications other than MG, and reports describing the development of MG as a paraneoplastic event were excluded. After the initial title and abstract screening, we assessed the full texts of potentially included studies and hand searched their reference lists to gather additional relevant publications. In support, an online search on Google Scholar was also performed using the same terms to collect any gray literature report. The entire literature search was conducted by two independent investigators (CT and P-PL), and the selection of retrieved reports, with the numbers of the records identified or excluded and the reasons of exclusions, is represented in **Figure 2**. According to the Preferred Reporting Items for Systematic Reviews and Meta-analyses (PRISMA) 2020 statement (http://www.prisma-statement.org), our flow diagram depicts the flow of information through the different phases of the screening process, mapping out the number of records identified, included, and excluded, and the reasons for exclusions (23). All available data on clinical and diagnostic features (e.g., neurological symptoms, imaging findings, serological analyses, and nerve conduction tests), management (e.g., pyridostigmine, prednisolone, intravenous immunoglobulin, plasmapheresis, and escalation to intensive care), and outcomes of these cases (e.g., resolution of myasthenic symptomatology, discontinuation or resumption of TKI, and cancer status) were collected. Data extraction was undertaken by two investigators (CT and P-PL) independently. The level of initial agreement was assessed using kappa statistics. Any discrepancy was resolved by a third independent investigator (DZ), who reviewed the data extraction.

## Literature Review Results

From the literature search, seven reports were identified (six case reports and one case series) that describe 12 cancer patients who developed MG after targeted therapy (15–21). The included articles were all retrospective studies, so the assessment of quality and risk of bias of analysis were performed using the 9-point Newcastle–Ottawa Scale (NOS) (**Table 1**). The agreement in eligibility evaluation and in data extraction was both high [kappa = 0.94 (0.90–0.98) and kappa = 0.96 (0.92–1.00)], respectively. **Table 1** summarizes all retrieved cases with the addition of our patient reported here. In total, these 13 cases had specific clinical signs of fatigable weakness but lacked consistent laboratory and electrodiagnostic findings corroborating the diagnosis; 5 of the 13 patients had tested positive for anti-AchR and 1 of the 13 for anti-MuSK antibodies, while serological testing was negative or not available for the rest of seven cases. Among them, two had elevated CPK, pointing the muscle involvement, and two had myopathic changes in the EMG, while CPK results and EMG data were not reported for the majority of patients. Nerve conduction studies, including RNS tests, were pathological in 10 of 13 cases. Coexistence of thymic abnormality was detected in one individual. Time of onset of myasthenic symptoms widely ranged from just 3 days to approximately 3 years of TKI administration. Standard therapeutic approach with pyridostigmine and prednisolone was initially administered to all patients, leading to MG resolution in five cases. IVIg was needed in only three cases, plasmapheresis in one case, and two required escalation to mechanical ventilation. It is clear that the high heterogeneity among the included studies (e.g., diverse underlying malignancies and different classes of targeted anticancer agents) and the small number of recorded neuromuscular events do not permit to further analyze and generalize with strong certainty the observations of this review. However, all identified cases with TKI-induced MG and their management are briefly presented and discussed below according to the type of administered kinase inhibitors (**Table 1**).

**Table 1:** Summary of cancer cases that developed seropositive (or not) TKI-induced MG.

Author, year (reference)	Type of TKI	Type of cancer, (setting)	No of patients	Age, (Sex)	Onset	Neurological symptoms (MG subtype)	Anti-AchR	Anti-MuSK	RNS/EMG	Serum CPK (U/L)	Treatment of MG	Discontinuation of TKI	Outcome of MG	NOS score
**BRAF and MEK (mitogen-activated protein kinase, MAPK) inhibitors**														
**Our presented case, 2021**	Dabrafenib and trametinib	BRAF-mutant melanoma, (metastatic)	1	57, (F)	3 years	Bilateral muscle weakness of upper arms and fatigability, dyspnea at exercise and dysphagia (generalized)	(−)	(+)	(+)/(−)	Normal	Initial MG manifestation: PG 20 mg t.i.d., Prednisone 20 mg qd	Yes, exacerbation of MG, after restarting of BRAF/MEK inhibitors and permanent discontinuation	Improvement	7
											Exacerbation of MG, one week after restarting of dabrafenib/trametinib: PG 20 mg t.i.d., Prednisone 25 mg qd IVIG twice weekly			
**Zaloum et al. (** 15 **)**	Dabrafenib and Trametinib	BRAF-mutant melanoma, (metastatic)	1	68, (M)	2 weeks	Bilateral ptosis, facial diparesis, nasal speech, proximal muscle weakness and fatigability, dysarthria, dysphagia, and dyspnea (generalized)	(+)	(−)	(−)/(−)	228	PG 30 mg q.i.d., Prednisone 100 mg QoD only for 2 doses due to sepsis,	Yes, reactivation of MG, after restarting of BRAF/MEK inhibitors and permanent discontinuation	Improvement within 24 h	7
											Reactivation of MG, after restart of dabrafenib/trametinib: PG 90 mg q.i.d., Prednisone 60 mg QoD 2 months later, concomitant IVIG twice weekly		New improvement after reactivation in a 4-week course	
**Alabdali et al. (** 16 **)**	Binimetinib	Low-grade serous ovarian cancer, (clinical trial after recurrence)	1	77, (F)	4 weeks	Head drop, cervical muscles fatigable weakness	(−)	N/A	(−)/(+)	Normal	PG 60 mg b.i.d., Prednisone 10 mg/day	Yes	Improvement within a few days	5
**Inhibitors of fused breakpoint cluster region and Abelson (BCR-ABL)**														
**Demichelis et al. (** 17 **)**	Imatinib	GIST (adjuvant)	1	65, (M)	1 week	Dysphagia, dysphonia, head drop, chewing, and swallowing difficulties, eye ptosis	(+)	(−)	N/A/ N/A	462	PG 60mg, q.i.d Plasmapheresis, after the second Plasma exchange session, acute respiratory failure and mechanical ventilation Methylprednisolone, 120 mg/day IVIg, 2g/kg one cycle	Temporally stopped for 2 days, but after subsequent resumption, the previous symptoms appeared again	Finally recovered after 1 month in ICU and a slow steroid tapering was followed for 1 month	6
**Kopp et al. (** 18 **)**	Imatinib	CML	1	40, (M)	3 months	Bilateral eye ptosis	(+)	N/A	(+)/ N/A	N/A	Pyridostigmine Prednisolone Thymectomy	No	Improvement	6
**Sanford et al. (** 19 **)**	Nilotinib	CML	1	40, (M)	6 months	Fluctuating diplopia and bilateral ptosis, proximal arm weakness, head flexion, and right deltoid fatigable weakness	(+)	N/A	(+)/ N/A	N/A	Pyridostigmine Prednisone	No, continuation of nilotinib	Resolution of his symptoms	6
**FMS-like tyrosine kinase 3 (FLT3) inhibitors**														
**Lehky et al. (** 20 **)**	Tandutinib and bevacizumab	Glioblastoma (clinical trial after recurrence)	6	60, (M)	14 days	Head drop, leg weakness	N/A	N/A	(+)/ N/A	N/A	N/A	Yes, tandutinib stopped	Improvement	6
				61, (M)	3 days	Head drop, leg weakness	(−)	N/A	(+)/ N/A	N/A		Yes, tandutinib stopped		
				50, (M)	10 days	Head drop, arm and leg weakness	(−)	N/A	(+)/ N/A	N/A		No		
				55, (M)	30d	Leg and arm weakness	N/A	N/A	(+)/ N/A	N/A		No		
				54, (M)	112d	Leg weakness	N/A	N/A	(+)/ N/A	N/A		No		
				60, (M)	63d	Leg weakness	N/A	N/A	(+)/ N/A	N/A		No		
**Anaplastic lymphoma kinase (ALK) inhibitors**														
**Desai et al. (** 21 **)**	Lorlatinib	NSCLC (metastatic, clinical trial after multiple recurrences)	1	58, (M)	3 months	Arm weakness, neck weakness, difficulty standing up and climbing stairs, bilateral ptosis and diplopia	(+)	(−)	(+)/(−)	Normal	PG, AZA (150 mg/day) and prednisone (30 mg/day alternating with 20 mg/day) intubation and plasma exchange during a crisis management	No	Improvement	7

MG, myasthenia gravis; TKI, tyrosine kinase inhibitors; AchR, acetylcholine receptor, RNS, repetitive nerve stimulation; EMG, electromyography; MuSK, muscle-specific kinase; GIST, gastrointestinal stromal tumor; CML, chronic myeloid leukemia; NSCLC, non-small cell lung adenocarcinoma; NOS, Newcastle–Ottawa Scale.

N/A; Not available.

### BRAF and MEK (Mitogen-Activated Protein Kinase) Inhibitors

#### Dabrafenib (Tafinlar^®^, GSK-2118436) and Trametinib (Mekinist^®^, GSK1120212)

Recently, Zaloum et al. reported another melanoma case who developed anti-AchR(+) MG after exposure to dabrafenib and trametinib, too. This patient was a 68-year-old man with BRAF V600-mutant melanoma of the scalp, which progressed with a parotid metastasis, 1 year after local excision of the primary tumor (15). After 2 weeks of targeted therapy, he was admitted with dysarthria, dysphagia, dyspnea, and fever. The physical examination showed bilateral ptosis, facial diparesis, nasal speech, proximal muscle weakness, and fatigability, suggesting a neuromuscular junction disorder. Laboratory testing revealed leukocytosis (20,100 × 10^3^/L) and mild elevation of creatinine phosphokinase (CPK, 228 U/L; reference range, 24–195 U/L), while chest imaging detected no evidence of thymoma but a right lower pulmonary consolidation, compatible with an aspiration pneumonia. Based only on the high clinical suspicion of myasthenic syndrome, the patient was empirically treated with pyridostigmine (30 mg, four times per day) and antibiotics, while BRAF/MEK inhibitors were temporally withheld. Within 24 h, his symptoms were substantially improved, and he was discharged 1 week later. At that time, there was only equivocal evidence of fatigable ptosis and proximal muscle weakness, and 2 Hz repetitive stimulation of the ulnar and facial nerves was normal. Over the next several weeks, he remained neurologically stable without pyridostigmine and restarted dabrafenib and trametinib. Within <2 months, symptomatology was relapsed (e.g., dysarthria, ptosis, and fatigable weakness), and the diagnosis of myasthenia was confirmed with positive anti-AchR antibodies in the serum, at a level of 6.45 nmol/L (reference range, ≤0.02 nmol/L). At this time, BRAF/MEK inhibitors were permanently discontinued, but the introduction of pyridostigmine and its increase to 90 mg four times per day produced only partial improvement. Prednisone and IVIg were needed to produce gradual amelioration and resolution of myasthenic symptoms over the following months.

#### Binimetinib (Mektovi^®^, ARRY-162)

Another recent case report described a 77-year-old woman with recurrent low-grade serous ovarian cancer who presented clinical and neurophysiological findings of MG, just 4 weeks after the initiation of binimetinib in a trial setting (16). The patient complained of indolently progressing, isolated head drop with no ocular, bulbar, or appendicular muscle weakness. The neurological examination showed localized muscle fatigue with repetitive neck flexion and extension in the cervical area. Blood tests (including CPK and anti-AchR antibodies), CT scans, needle EMG of the four extremities and of the paraspinal muscles, and nerve conduction studies generated unremarkable results. Only the single-fiber EMG was abnormal, demonstrating an impaired muscular reaction. The MEK inhibitor, binimetinib, was discontinued and neurological symptoms began improving within a few days. Further improvement was observed after the addition of pyridostigmine 60 mg twice daily, and myasthenic symptomatology was completely resolved with a daily dose of prednisone at 10 mg. The time frame of events, the clinical presentation, and the response to standard antimyasthenic treatment were consistent with a drug-induced MG, as a consequence of exposure to MEK inhibitor (16).

### Inhibitors of Fused Breakpoint Cluster Region and Abelson

#### Imatinib (Glivec^®^, STI571, CGP57148B)

In this class of TKIs, Demichelis et al. reported a case of a 65-year-old man with a gastrointestinal stromal tumor (GIST) who commenced imatinib on adjuvant setting following an initial resection of the tumor (17). A few days after initiation of targeted therapy, the patient presented with dysphagia and weakness of the neck extensor muscle, which subsided after a 2-day pause of TKI. Two days after the reinitiation of imatinib, the previous symptoms relapsed with additional right eyelid ptosis, head drop, and dysphonia. The whole-body imaging did not detect thymic disorder or GIST metastases, while CPK was elevated (462 U/L; reference range, 24–195 U/L). The patient was empirically treated with pyridostigmine and plasmapheresis, but after the second plasma exchange session, he developed acute respiratory failure and required escalation in intensive care unit and mechanical ventilation. The diagnosis of MG was confirmed by the detection of anti-AchR and anti-titin antibodies in the serum. Subsequent clinical improvement was observed following IVIg administration, and after 1 month of intensive rehabilitation, neurological symptoms were completely resolved. Authors hypothesized a drug-induced immune-mediated event due to the temporal pattern of MG manifestations.

After 3 months of treatment with imatinib, another case of a 40-year-old man with CML, who complained of bilateral ptosis as his sole symptom, was presented (18). Abnormal RNS test and positive anti-AchR antibodies were consistent with MG diagnosis, and the patient was treated with pyridostigmine and prednisolone. However, subsequent chest CT revealed the coexistence of a mediastinal mass, which was resected, and the histopathological analysis revealed thymic hyperplasia. The surgical intervention led to resolution of the symptoms with no future recurrence. Although there was obvious thymoma-associated origin of the myasthenic syndrome, authors proposed that the exposure to imatinib accelerated the onset of MG symptomatology.

#### Nilotinib (Tasigna^®^, AMN107)

Being treated for 6 months with nilotinib, a second-generation TKI of the same class for CML, a 40-year-old man developed bilateral ptosis, diplopia, and proximal arm weakness (19). Interestingly, after 3 months of TKI treatment, the patient had already experienced complete hematological response, with no documented side effect. When his complaints started, an otherwise unremarkable neurological examination detected only a mild weakness on head flexion and right deltoid muscle, while the ice pack test was rendered positive. Imaging tests were normal, but anti-AchR antibodies were positive, and RNS revealed attenuated responses in the facial and spinal accessory nerves confirming the diagnosis of seropositive generalized MG. The patient commenced pyridostigmine plus prednisone, and his symptoms were improved. Given the favorable response to nilotinib, the continuation of TKI was decided. At the time of publication, the authors were still uncertain if nilotinib triggered the onset of myasthenic symptoms and if the cessation of TKI would speed their resolution. Of interest, Gundin and colleagues reported a similar to MG disorder but with multiple cranial nerve involvement in a male patient with CML treated with nilotinib (24). The patient reported dysgeusia and blurred and double vision as result of internal rectus paralysis in the eyes and variation of two pairs of cranial nerves. Based only on clinical criteria, he was diagnosed of ocular and bulbar myasthenic syndrome, and a dose reduction from 400 to 300 mg twice daily was employed. The patient remained in complete molecular remission with less dosage than usual.

### FMS-Like Tyrosine Kinase 3 Inhibitors

#### Tandutinib (MLN518)

In a case series, Lehky et al. reported six patients with recurrent glioblastoma, who were treated with tandutinib and bevacizumab in a clinical trial setting and developed clinical and electrophysiological findings consistent with MG (20). All subjects, without any prior history of neurological disease, presented reversible facial, neck, and proximal limb weakness soon after the regimen initiation, with half of them in the first 2 weeks. RNS testing showed decremental responses in all patients but improved or even normalized in evaluated patients post-tandutinib discontinuation. Four patients had short-duration motor potentials in needle EMG, showing neuromuscular blockade or myopathy (e.g., due to dexamethasone for vasogenic edema control). Single-fiber EMG was performed in three patients, with abnormal findings. Antibodies against AchR, striated muscle, and VGCC were examined in two of six patients but were not detected in either. Anti-MuSK was evaluated in one patient but was also negative. Given the absence of previous association of bevacizumab with neuromuscular dysfunction and the relatively rapid onset of symptoms following the initiation of TKI, the authors highlighted tandutinib as the causative factor behind the myasthenic syndrome and proposed that this TKI has a direct “off-target” effect on the neuromuscular junction, interfering with postsynaptic targets rather than generating an immune-mediated response. Moreover, the clinical improvement and the normalization of available neurophysiological tests were coupled with tandutinib reduction or discontinuation, supporting a dose-dependent correlation between the MG symptomatology and this TKI agent. Most of the patients tolerated sufficiently the reintroduction of tandutinib at lower dose, while permanent discontinuation of TKI was decided in two patients, considering the risk of myasthenia recurrence.

### Anaplastic Lymphoma Kinase Inhibitors

#### Lorlatinib (Lorbrena^®^, Lorviqua^®^)

Desai et al. presented a heavily pretreated 56-year-old man with stage IV non-small cell lung adenocarcinoma, who developed MG being on partial response after 3 months of treatment with lorlatinib (PF-06463922, 100 mg daily) (21). Before this ALK TKI with high activity in patients harboring G1202R resistant mutation to crizotinib and ceritinib, the patient had initially received chemotherapy and, sequentially, crizotinib and ceritinib after the identification of ALK rearrangement. His neurological disorder was presented with weakness in his arms followed by difficulties in standing from a sitting position, in climbing stairs, and in holding his head up. His symptoms were gradually worsened covering the whole daytime, and in parallel, he developed bilateral ptosis and diplopia at the end of the day. Finally, he required admission to the emergency room with dyspnea and oxygen desaturation. The diagnosis of MG was confirmed by an abnormal RNS test but normal needle examination and serum CPK. Circulating AchR antibodies (13.4 nmol/L; normal ≤0.02 nmol/L), AchR-modulating antibodies (100%; normal <20%), and striated muscle antibodies (1:1,920; normal <1:120) were elevated, while MuSK and VGCC antibodies were negative, and no evidence of thymoma was identified. Shortly after the initiation of pyridostigmine, almost complete clinical recovery was achieved, and lorlatinib treatment was resumed. After that, he had one episode of respiratory crisis requiring intubation responding to plasma exchange. His immunosuppression was intensified with azathioprine (150 mg/day) and prednisone (30 mg/day alternating with 20 mg/day) in addition to pyridostigmine. Despite the concurrent MG, the patient had limited therapeutic options and remained on lorlatinib at a lower dosage of 50 mg daily, keeping under control his lung cancer. At the time of publication, his clinical symptomatology and his laboratory findings (positive AchR binding and modulating antibodies, 0.64 nmol/L and 89%, respectively, and positive striational antibodies 1:1,920) remained consistent with MG. The authors recognized an association between the onset of disorder and the ALK inhibition, since 5 years before any targeted therapy, AchR antibodies were negative while striational antibodies were positive (1:15,360). Given that the patient had no myasthenic symptoms over the previous 7 years of his NSCLC course, even while treated with older generation TKIs of the same class, lorlatinib seems to be the most possible cause of worsening neuromuscular symptoms (21).

## Discussion

Many drugs of everyday practice have been found capable of inducing neuromuscular dysfunction or exacerbating a smoldering myasthenic syndrome in case reports, but only few targeted anticancer agents were included (3, 7, 25). Here, we presented the first case with anti-MuSK(+)MG after long-term exposure to BRAF/MEK inhibitors, where the reactivation of myasthenia after dabrafenib/trametinib rechallenge further supports that the TKI association may be more than coincidental. The following literature review identified additional 12 cancer cases with TKI-related MG reported in seven articles, mainly case reports and one case series. In most of them, the myasthenic symptomatology clearly corresponded to the TKI discontinuation and to the standard treatment with pyridostigmine (acetylcholinesterase inhibitor, to increase the available amount of Ach at the neuromuscular junction) and prednisolone (to suppress the enhanced autoimmunity).

The exact mechanism by which TKIs could contribute to the development of unprecedented neuromuscular disorder or to the exacerbation of a subclinical pre-existing myasthenia is unknown. In general, TKI-induced MG can be divided into (i) immune mediated, when the autoimmune reaction, triggered by a TKI, turned against postsynaptic membrane antigens or into (ii) non-immune mediated, non-antigen specific, myasthenic syndrome, following a more direct “off-target” effect of the drug on tyrosine kinase domains in the neuromuscular junction. Beginning from the last pathogenic mechanism, some interesting insights are given by Lehky et al. and by preliminary phase I findings (20, 26). The MuSK protein is a transmembrane component of the postsynaptic muscle endplate that mediates into the clustering of AchR and maintains the integrity of the neuromuscular junction (27). Containing functional tyrosine kinase domains on the cytoplasmic surface, MuSK protein may be susceptible to “off-target” inhibition by multitargeted TKIs. For example, tandutinib can target platelet-derived growth factor (PDGF) receptor, which colocalizes with the AchR at the postsynaptic membrane, while breakpoint cluster region and Abelson (BCR-ABL) inhibitors, such as imatinib, nilotinib, and dasatinib, can target protein sequences within the ATP-phosphate binding loop with close relation and unknown interaction to other TKI receptor such as MuSK (28). MuSK inhibition hinders DOK7 phosphorylation and halts the AchR clustering cascade, impairing junctional transmission (29). In fact, in patients with acute myelogenous leukemia or myelodysplastic syndromes, the primary tandutinib dose-limiting toxicity was generalized muscle weakness and fatigue when doses reached the range between 525 and 700 mg twice a day (26). Other possible mechanisms by which BRAF/MEK inhibitors or FLT-3 inhibitors could unmask pre-existing subclinical MG are alteration of the structure, the stability, or the function of the neuromuscular junction (15).

Regarding the potential immune-mediated processes, the administration of a TKI could disrupt normal immune surveillance and enhance autoimmunity *via* reducing regulatory T (Treg) cell-mediated suppressive activity, driven by forkhead box protein 3 (FOXP3) transcription factor (30). For instance, no differences were found in the subpopulations of CD4^+^CD25^+^ Tregs between patients with MG and healthy controls, but there are significantly lower presence and lower fluorescence intensity of FOXP3 within CD4^+^CD25^+^ Tregs in MG patients (31, 32). Following treatment with a TKI, the number of total T cells, Tregs, CD4^+^ T and CD8^+^ T cells were decreased to various degrees and more significantly with time, while specific interactions were also noted in the expression of FOXP3 (33). Imatinib, dasatinib, and nilotinib demonstrated similar inhibitory effects on Tregs *in vivo*; imatinib and dasatinib produced more marked effects compared to nilotinib on the function of Tregs *in vitro*. Indeed, in these treatments, suppressive cytokines [interleukin (IL)-4, IL-10, and transforming growth factor beta (TGF-β)] and molecules (FOXP3, GITR, and CTLA-4) were significantly limited for up to 6 months after TKI initiation (34, 35). Instead, nilotinib could downregulate the expression of FOXP3 only when administered at higher doses (35). According to *in vitro* and *in vivo* data, the FOXP3 expression is negatively affected by the impaired STAT3 and STAT5 phosphorylation upon imatinib treatment (36, 37). Similarly to BCR-ABL inhibition, blocking of the MEK/ERK pathway was found to delay the maturation of resting Tregs (rTregs) to active Tregs (aTregs) and to significantly decrease the percentage of FOXP3+rTregs and FOXP3+aTregs (38). Both cases of Zaloum et al. and Maurice et al. have described the downregulation of FOXP3 expression on Treg, pinpointing an immunomodulatory property of MEK inhibitors (39, 40). Interestingly, in the study of Lieske et al., among the eight different MEK inhibitors evaluated for their potency in interfering FOXP3 expression, trametinib and binimetinib were emerged as the most active (40). A more recent study noted another immune-mediated mechanism of action for imatinib (41). More specifically, imatinib was found to increase apoptosis in aTregs but only moderately on rTregs (41). The T-cell receptor (TCR)-dependent proliferative status of aTregs renders them more susceptible to signal-deprived apoptosis induced by imatinib (42). Previous data have also detected this “off-target” effect of imatinib on early TCR/CD28 signaling (43).

Over and above the drug-induced etiology of myasthenia, the paraneoplastic possibility should always be considered. A great example of paraneoplastic myasthenic event is the Lambert–Eaton myasthenic syndrome (LEMS), caused by autoantibodies against VGCC on presynaptic nerve terminals that impair the release of Ach. This autoimmune disorder is diagnosed clinically on the basis of a triad of symptoms (proximal muscle weakness, hyporeflexia, and autonomic disturbance), supported by electrophysiological findings and the presence of anti-VGCC autoantibodies (44). Between 40% and 62% of patients diagnosed with LEMS are found to have small-cell lung cancer, almost all of whom develop neurological symptoms before their cancer diagnosis (44). The timely identification of LEMS and appropriate screening for SCLC could improve the outcome of both conditions. In case of MG, this neuromuscular disorder was reported as a paraneoplastic event in two patients with GISTs, in two patients with CML [beyond the abovementioned cases treated with imatinib (17–19)], in three patients with ovarian carcinoma, and in five patients with NSCLC (all these additional references are provided as a supplementary file). On the other side, three reports were also recognized describing remission of myasthenic symptomatology after TKI administration for renal cell carcinoma (45), CML (46), and postpolycythemia vera (PV) myelofibrosis (47). However, in the first two cases, the improvement of symptoms was secondary to cancer response, and in the context of PV, MG could be a paraneoplastic manifestation. Focusing further on the patient with post-PV myelofibrosis, the complete serological and symptomatic remission of his myasthenia was achieved after treatment with a JAK1/2 inhibitor, ruxolitinib (47). Of interest, ruxolitinib and some other Janus family tyrosine kinases (JAK) inhibitors, already approved for refractory dermatomyositis, alopecia areata, and rheumatoid arthritis, are currently under investigation for the treatment of MG (48). Serum cytokines are strongly implicated in the pathogenesis of MG, and JAK inhibitors are blocking crucial pathways of interleukin production. For example, IL-17 levels were higher and significantly correlated with the anti-AchR titers in generalized MG (49, 50), while IL-6 is dominant in any autoimmune response, and its downstream signaling involves a sequence of three JAK catalyzed phosphorylations (51). In the absence of IL-6 or blocking of its signaling, no other cytokine can substitute it in the conversion of conventional T cells into FOXP3(+)T-regs. Therefore, ruxolitinib that potently inhibits JAK1 and JAK2 kinases of IL-6, and tocilizumab, an anti-IL-6 humanized monoclonal antibody, have been reasonably studied and found to be effective in cases of myasthenia refractory to rituximab (52). Another JAK1 and JAK3 inhibitor, tofacitinib, is also under investigation by a phase I trial (NCT04431895) in refractory MG (48).

In conclusion, the emergence of MG after administration of targeted anticancer agents is occasionally reported in the literature. Thus, these medications cannot significantly influence the already low incidence (0.3–2.8 per 100,000) and prevalence (5.35–35 per 100,000) of MG, and counting their clinical benefit, there is no clear contraindication of using them (7, 53). However, physicians and healthcare providers should be aware of such rare but possible neurological complications following TKI administration and should also be on alert for the need of a multidisciplinary approach and a multimodality treatment. A good rule of thumb is to assume that any newly introduced anticancer drug, including targeted therapy or immunotherapy, may trigger myasthenic symptomatology, and each individual should be closely monitored for such symptoms (6). In case of clinical deterioration, TKI should be withdrawn, and standard antimyasthenic therapy should be initiated. Given the limited therapeutic options and the marked efficacy of novel targeted agents, the decision of TKI rechallenge is not so straightforward but should be reconsidered in a multidisciplinary level. For instance, in our case when the melanoma relapses, a re-exposure to BRAF/MEK inhibitors should be individually discussed after weighing the risk–benefit ratio, as this decision could not be supported by the current evidence. Following the increased use of targeted therapies for various cancers and the upcoming combinations with immune-checkpoint inhibitors, more immune-mediated and not-immune-mediated neurological and neuromuscular adverse events will come to light, requesting for personalized treatment. The early diagnosis of such disorders, the adaptation of oncological therapy, and the optimized management are essential to extend the on-treatment and overall survival of TKI-treated cancer patients.

## Data Availability Statement

The datasets presented in this study can be found in online repositories. The names of the repository/repositories and accession number(s) can be found in the article/supplementary material.

## Ethics Statement

The studies involving human participants were reviewed and approved by Medical Ethics Committee of University-affiliated Laikon General Hospital, School of Medicine, National and Kapodistrian University of Athens, Greece. The patients/participants provided their written informed consent to participate in this study. Written informed consent was obtained from the individual(s) for the publication of any potentially identifiable images or data included in this article.

## Author Contributions

DZ, HG, DM, and SB treated the presented case. CT, P-PL, and DZ reviewed the literature. DZ, DM, PA, and HG wrote the manuscript. All authors contributed to the article and approved the submitted version.

## Conflict of Interest

HG has received grants and personal fees from Roche, BMS, MSD, and Novartis and personal fees from Amgen and Pierre Fabre, outside the submitted work. PA has a consultant/advisory role for BMS, Roche-Genentech, MSD, Array, Novartis, Merck Serono, Pierre Fabre, Incyte, NewLink Genetics, Genmab, Medimmune, AstraZeneca, Syndax, SunPharma, Sanofi, Idera, Ultimovacs, Sandoz, Immunocore, 4SC, Alkermes, Italfarmaco, Nektar, Boehringer-Ingelheim, Eisai, Regeneron, Daiichi Sankyo, Pfizer, Oncosec, Nouscom, Takis, Lunaphore, and Seagen, and he has also received research funds from BMS, Roche, Array, and Sanofi and travel support from MSD, outside the submitted work.

The remaining authors declare that the research was conducted in the absence of any commercial or financial relationships that could be construed as a potential conflict of interest.

## Publisher’s Note

All claims expressed in this article are solely those of the authors and do not necessarily represent those of their affiliated organizations, or those of the publisher, the editors and the reviewers. Any product that may be evaluated in this article, or claim that may be made by its manufacturer, is not guaranteed or endorsed by the publisher.
